# Important role of the right hemisphere in post-stroke cognitive impairment: a functional near-infrared spectroscopy study

**DOI:** 10.1117/1.NPh.12.1.015008

**Published:** 2025-02-17

**Authors:** Yinan Ai, Yu Zhang, Fang Zheng, Haojie Hu, Mingyu Yin, Ziying Ye, Haiqing Zheng, Liying Zhang, Xiquan Hu

**Affiliations:** a The Third Affiliated Hospital, Sun Yat-sen University, Department of Rehabilitation Medicine, Guangzhou, China; b New York University, Department of Psychology, College of Arts and Sciences, New York, New York, United States

**Keywords:** post-stroke cognitive impairment, functional connectivity, functional near-infrared spectroscopy, stroke, aphasia

## Abstract

**Significance:**

The current neuromodulation treatment for post-stroke cognitive impairment (PSCI) is formulated based on interhemispheric inhibition, which is particularly relevant in the context of motor disorders after stroke. However, the pathological mechanism of PSCI remains unclear, which is completely different from motor disorders. Therefore, exploring the pathological brain characteristics of PSCI can provide a reliable theoretical basis for effective neuromodulation treatment for it.

**Aim:**

We explored different functional connectivity (FC) manifestations of PSCI with or without aphasia via functional near-infrared spectroscopy (fNIRS) to provide a pathological basis for the neuromodulation strategy.

**Approach:**

We collected cognitive performance and fNIRS data from patients with PSCI without aphasia (PSCI group, n=33) and patients with post-stroke aphasia (PSA group, n=31), using normal cognition stroke patients (SC group, n=32) and healthy subjects (HC group, n=31) as controls. Differences in FC among different types of stroke-related cognitive impairment were analyzed.

**Results:**

The overall FC in the PSCI group was lower than that in the SC or HC group, and the FCs of the right hemisphere, the right default mode network (DMN), and the right central executive network (CEN) of PSCI patients were significantly lower than those of the left ones. In the PSA group, the FCs of the DMN and CEN were not lower than those in the SC and HC groups, and the FC of the left hemisphere was significantly greater than that of the right hemisphere. In addition, the FC of PSCI patients with right lesions was weaker than that of left lesions, which was closely correlated with the cognitive scale.

**Conclusions:**

Unlike the left hemisphere activation strategy commonly used previously, our results suggest that the important role of the right hemisphere may be overlooked in PSCI patients with or without aphasia. Future treatment options and studies could consider focusing on the right hemisphere or bilateral hemispheres.

## 1 Introduction

About 40% to 70% of stroke survivors still have various degrees of cognitive impairment within 6 months after the onset of stroke,^1^ which is often accompanied by aphasia.^2^ Non-invasive brain stimulation (NIBS), a common technique for neuromodulation therapy, is an important new means of treating post-stroke dysfunction.^3^^,^^4^ NIBS can act on specific brain areas non-invasively and play a neuromodulation role by enhancing or inhibiting the nerve excitability of the brain regions, to improve the corresponding cognitive or motor functions of subjects.^3^ The target and parameter selection of NIBS are the keys to its efficacy, but the optimal treatment plan is still controversial.^5^ The optimal NIBS protocol for post-stroke cognitive impairment (PSCI) is not mentioned in the guidelines. In the current NIBS guidelines for aphasia patients, the stimulation mostly targets at the brain regions corresponding to language (Broca’s or Wernicke’s area), and left hemisphere excitation or right hemisphere inhibition is the main stimulus prescription,^6^^–^^8^ which is mainly formulated based on interhemispheric inhibition (IHI), given that most individuals are left hemisphere dominant.^9^^,^^10^ Although IHI is particularly relevant in the context of motor disorders, its applicability to cognitive and language functions remains uncertain. Furthermore, there is still a lack of evidence for pathological functional changes in PSCI. Tailoring personalized NIBS strategies, which take into account the distinctive pathological changes in brain functional connectivity (FC) observed in patients with PSCI and post-stroke aphasia (PSA), may hold the key to achieving a breakthrough in enhancing the current efficacy of NIBS.

Functional recovery after stroke is strongly correlated with changes in brain FC,^11^^–^^13^ and the status of the resting-state brain network can help predict the rehabilitation prognosis of stroke patients^14^ and guide rehabilitation treatment plans. However, functional magnetic resonance imaging (fMRI) exhibits limited resistance to motion and electromagnetic interference. Patients with cognitive impairment have poor adherence, often moving frequently during fMRI to the detriments of data quality, and the use of sedatives may affect the brain state. In addition, patients with metal implants could not undergo fMRI, which greatly limited the scope of enrolled patients and made the study results biased. Functional near-infrared spectroscopy (fNIRS) imaging technology can effectively detect changes in oxyhemoglobin (HBO) in the cerebral cortex based on the different absorption rates of HBO in near-infrared light with different wavelengths.^15^^,^^16^ Studies have shown that the ability of fNIRS to detect hemodynamic correlates of neural activity is comparable to that of the fMRI-BOLD.^17^ Given its convenient operation, wide application range, strong anti-interference ability, and low cost, fNIRS is currently an ideal replacement for fMRI-BOLD,^18^ which avoids the shortcomings of fMRI above.

To identify better neuromodulatory prescriptions for PSCI and PSA patients, we analyzed the FC of brain networks in PSCI patients with or without aphasia using fNIRS. Stroke patients with normal cognition and healthy volunteers of the same age were used as controls to explore the pathological changes in the brain networks of PSCI patients. The aim of this study was to provide a reliable basis for the target and parameter setting of neuromodulation methods for stroke.

## 2 Methods

### 2.1 Participants

This study was a case-control study with participants selected from the baseline data of two large randomized controlled studies (ChiCTR2200062037, ChiCTR2300074898), which were approved by the Third Affiliated Hospital of Sun Yat-sen University ethics committee. A total of 127 subjects were enrolled in this study, all of whom were stroke patients or family members who were admitted to the Rehabilitation Department of the Third Affiliated Hospital of Sun Yat-Sen University from February 2023 to February 2024 and aged between 35 and 75 years, including 33 patients with cognitive impairment without aphasia after stroke (PSCI group), 31 patients with aphasia after stroke (PSA group), 32 patients with normal cognition after stroke (SC group), and 31 healthy subjects of the same age (HC group). The patient inclusion criteria were a single-event stroke, post-stroke duration of longer than 2 weeks and <12 months, age between 35 and 75 years, and previously right-handed. The presence of cognitive impairment was confirmed using the Mini-Mental State Examination (MMSE≤25), and Western Aphasia Battery (WAB) scores were used to indicate patients without aphasia (aphasia quotient [AQ]≥93.8) or with aphasia (AQ<93.8). In stroke patients with normal cognition and language, both WAB and MMSE scores were within the normal range. We excluded patients with global aphasia who were unable to complete the scale assessment, patients with skull deficiency who were unable to complete the fNIRS, and patients with severe neuropsychiatric disorders or affective disorders that could affect the test results. Healthy subjects were required to have no history of central nervous system injury or related diseases. All the subjects participated voluntarily and signed informed consent forms.

### 2.2 Behavioral Outcomes

All participants were assessed on two scales for overall cognitive function, the MMSE and the Montreal Cognitive Assessment Scale (MoCA), which include executive function, orientation, calculation, language ability, delayed recall, abstract thinking, and attention. Patients with PSA were assessed by the WAB. Considering the bias caused by motor function in brain networks, we evaluated the functional activities of patients’ upper extremities and wrists using the Fugl-Meyer Assessment Upper Extremity (FMA-UE) scale to reflect patients’ motor ability. Each subject was evaluated by a trained rehabilitation therapist.

### 2.3 Functional Near-Infrared Spectroscopy

#### 2.3.1 Data acquisition

We collected 5 min of resting-state fNIRS data for each subject, during which the patients were asked to rest with their eyes closed, remain still, and try not to think, in a quiet, separate room with the lights off. In this experiment, NirScan-9000A equipment (Danyang Huichuang Medical Equipment Co., Ltd., China) was used to continuously measure and record the concentration changes of brain oxygenated hemoglobin (HbO) and deoxyhemoglobin (HbR) during the task. The system consists of near-infrared light sources (light emitting diodes) and avalanche photodiodes as detectors, with wavelengths of 730, 808, and 850 nm and a sampling rate of 11 Hz. The experiment uses 14 light sources and 14 detectors to form 35 effective channels. The average distance between the source and the detector is 3 cm (range 2.7 to 3.3 cm), with reference to the Brodmann and LPBA40.^19^ The brain area mainly observed in this study is the frontal and parietal lobes.

#### 2.3.2 Data preprocessing

**Fig. 1:**
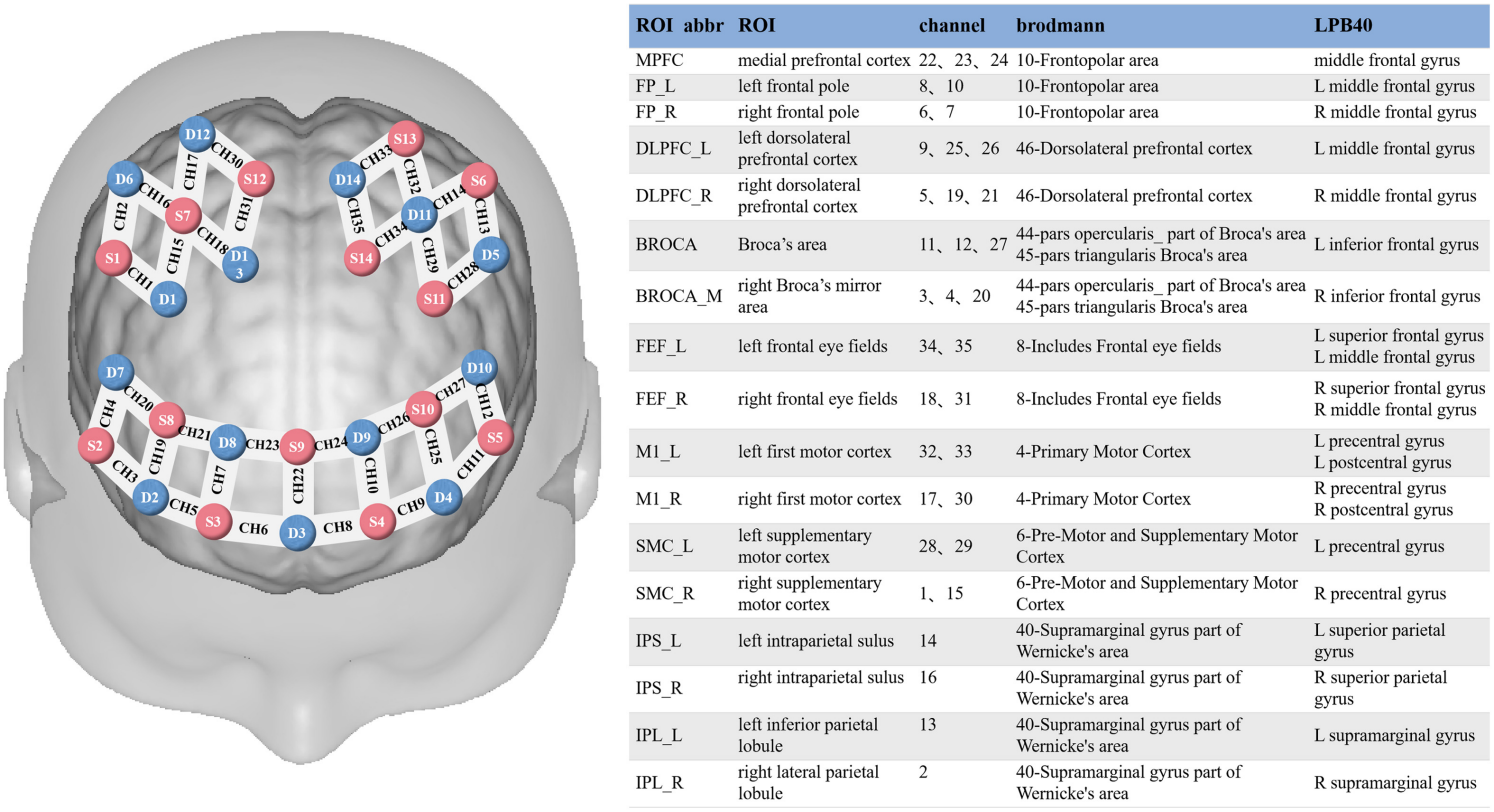
Distribution of fNIRS channels in the brain.

The NirSpark software (HuiChuang, China) package, which has been used in previous experiments, was used to preprocess fNIRS signals. Detailed preprocessing procedures can be found in the Supplementary Material. The FC between all 35 channels was defined as the overall FC of the brain. We combined the Brodmann and LPBA40 brain maps to divide the 35 channels into 17 regions of interest (ROIs), which were shown in Fig. 1. The average value of the FCs among the MPFC, IPL_L, and IPL_R included in the default mode network (DMN) was used to represent the FC of the DMN (DMN-FC). The mean FC of the bilateral dorsomedial prefrontal cortex (DLPFC) and bilateral IPS contained in the central executive network (CEN) was used to represent the FC of the CEN (CEN-FC). The FC of all 35 channels was used to reflect the FC situation of the overall brain (overall FC). Other brain regions or networks that are closely related to cognition or language are barely detectable on our fNIRS effective channels, so we did not analyze them. Pearson correlation was used in the FC analysis, and false discovery rate (FDR) correction was performed for all FC values. In addition, we calculated the number of connected edges of each network of interest with a threshold value of 0.5.

#### 2.3.3 Data analysis

We first conducted ANOVA of the differences between groups. An α<0.05 was considered to indicate statistical significance. For indicators with significance or marginal significance, we conducted post hoc comparisons of multiple groups, and Bonferroni correction was performed for P values. For multiple comparisons of channels and ROIs, we used FDR correction.

## 3 Results

### 3.1 General Functions and Characteristics

The baseline demographic and clinical characteristics of the study participants are shown in Table 1. The enrolled individuals had a mean (SD) age of 61.4 (9.3) years and included 97 men (76.4%); the average time since stroke onset was 8.4 (5.4) weeks. There were no significant differences in age or sex among the four groups. In addition, there were no significant differences in disease time, MRI lesion location (cortical or subcortical), or motor performance among the three groups with stroke.

**Table 1:** Characteristics of the subjects.

	PSCI	PSA	SC	HC	p
**Age [95%CI]**	63.79 [60.99 66.58]	58.13 [54.31 61.95]	62.09 [58.88 65.31]	58.74 [54.65 62.84]	0.062
**Sex (%)**	25(75.8)/8(24.3)	24(77.4)/7(22.6)	28(87.5)/4(12.5)	20(64.5)/11(35.5)	0.200
**Course (week) [95%CI]**	7.86 [6.18 9.55]	9.55 [7.19 11.90]	7.97 [6.23 9.71]	—	0.381
**MRI lesions (%)**					0.344
Subcortical	19(57.6)	17(54.8)	13(40.6)	—	
Cortical	14(42.4)	14(45.2)	19(59.4)	—	
**Lesion side (%)**					<0.001*
Left lesions	6(18.2)	31(100)	14(43.8)	—	
Right lesions	27(81.8)	0(0)	18(56.3)	—	
**MoCA [95%CI]**	9.52 [7.95 11.08]	8.32 [6.49 10.16]	20.47 [19.18 21.76]	26.61 [25.97 27.26]	<0.001*
**MMSE [95%CI]**	15.12 [13.16 17.09]	14.94 [13.14 16.73]	27.13 [26.69 27.56]	28.65 [28.17 29.12]	<0.001*
**WAB [95%CI]**	—	67.66 [60.87 74.46]	—	—	—
**FMA [95%CI]**	26.27 [19.44 33.11]	35.68 [26.26 45.10]	31.41 [24.11 38.71]	—	0.231

* and bold represent p<0.05

In terms of behavioral scales, there were significant differences in MMSE (p<0.001) and MoCA (p<0.001) scores among the four groups. However, there was no significant difference in MMSE ($p_{a}=1$) or MoCA ($p_{a}=1$) scores between PSCI patients and PSA patients. There was no significant difference in MMSE scores between the SC and HC groups ($p_{a}=0.701$) after Bonferroni correction, but MoCA scores in the SC group were significantly lower than those in the HC group ($p_{a}<0.001$). In terms of motor function, there was no significant difference in FMA-UE scores among the three-stroke groups (p=0.231).

### 3.2 Specific Changes in FC among Stroke Patients with Different Dysfunctions

**Fig. 2:**
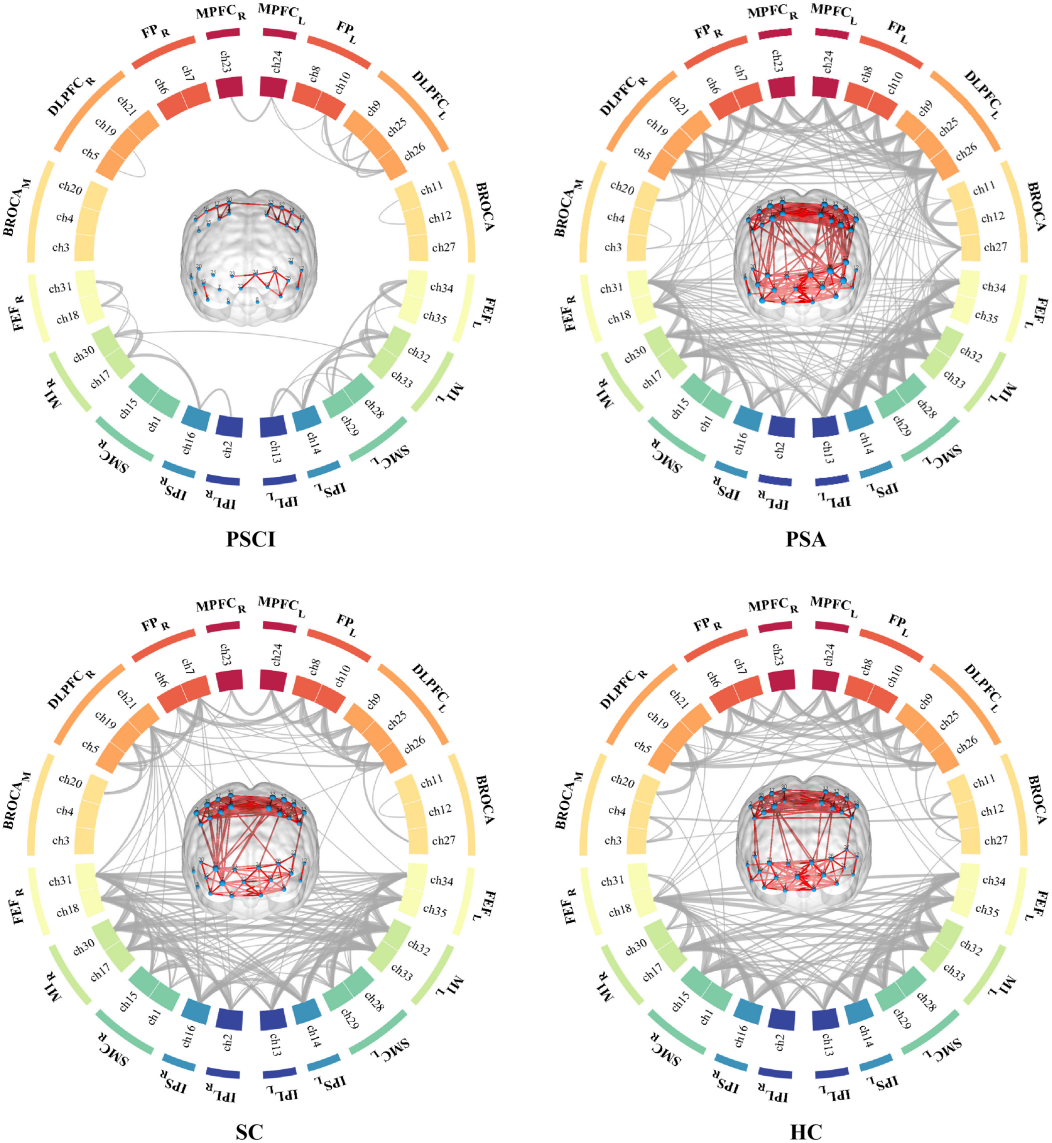
FC of patients in four different groups. The FC between each channel or ROI represented by gray lines of varying thickness in a circle map. The thickness of the lines corresponds to the strength of the FC. Both the circle map and the brain map showed an FC threshold of 0.5.

**Fig. 3:**
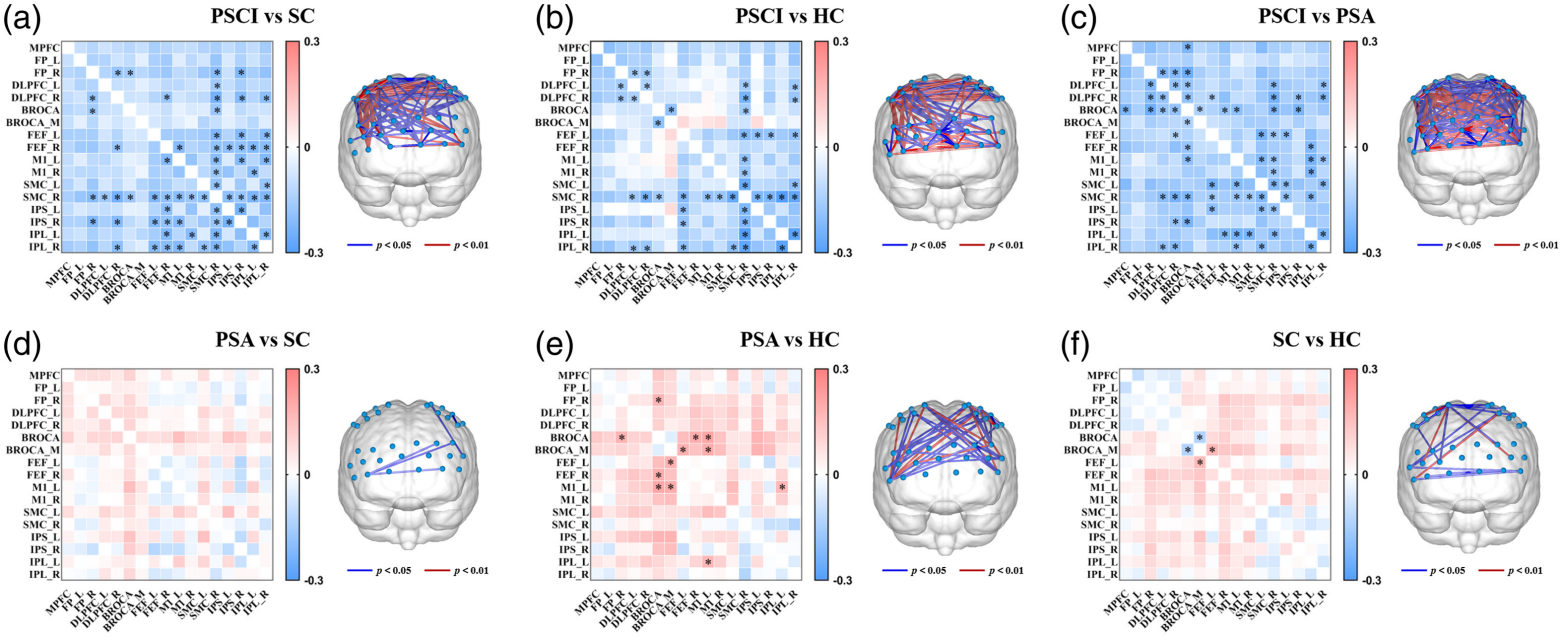
Post hoc comparison of FC in ROIs and channels after FDR in four groups of patients. *p<0.05.

There was a significant difference in overall FC among the four groups (p<0.001), and the graph theory analysis results at a threshold of 0.5 were also similar (p<0.001) (Fig. 2). After ROI FDR correction, there were still significant differences in many connections between each ROI. We conducted post hoc comparisons between groups of the connections with differences and performed FDR correction again. The results are shown in Fig. 3. There were several significant differences in the FC of the ROIs between the PSCI and SC groups. In particular, the FCs between the right frontal cortex and right parietal cortex and between these two regions and other brain regions in the PSCI group were significantly weaker than those in the SC group [Fig. 3(a)]; the difference between the PSCI and HC groups was similar to the difference between the PSCI and SC groups [Fig. 3(b)], and different FCs existed mainly between the right frontal and parietal cortex and between these regions and the contralateral hemisphere. The whole-brain FC of PSCI patients was significantly lower than that of PSA patients [Fig. 3(c)]. Compared with those of the SC group, the ROI FCs of the PSA group only showed an increasing trend, but there was no significant difference in the ROIs [Fig. 3(d)]. Compared with those of HCs, the FCs of PSA patients increased, mainly in the bilateral frontal lobes, especially the FC between Broca’s area and the mirror Broca’s area [Fig. 3(e)]. For SC patients, the FCs between Broca’s area and the mirror Broca’s area were weaker than those of HC subjects [Fig. 3(f)].

**Fig. 4:**
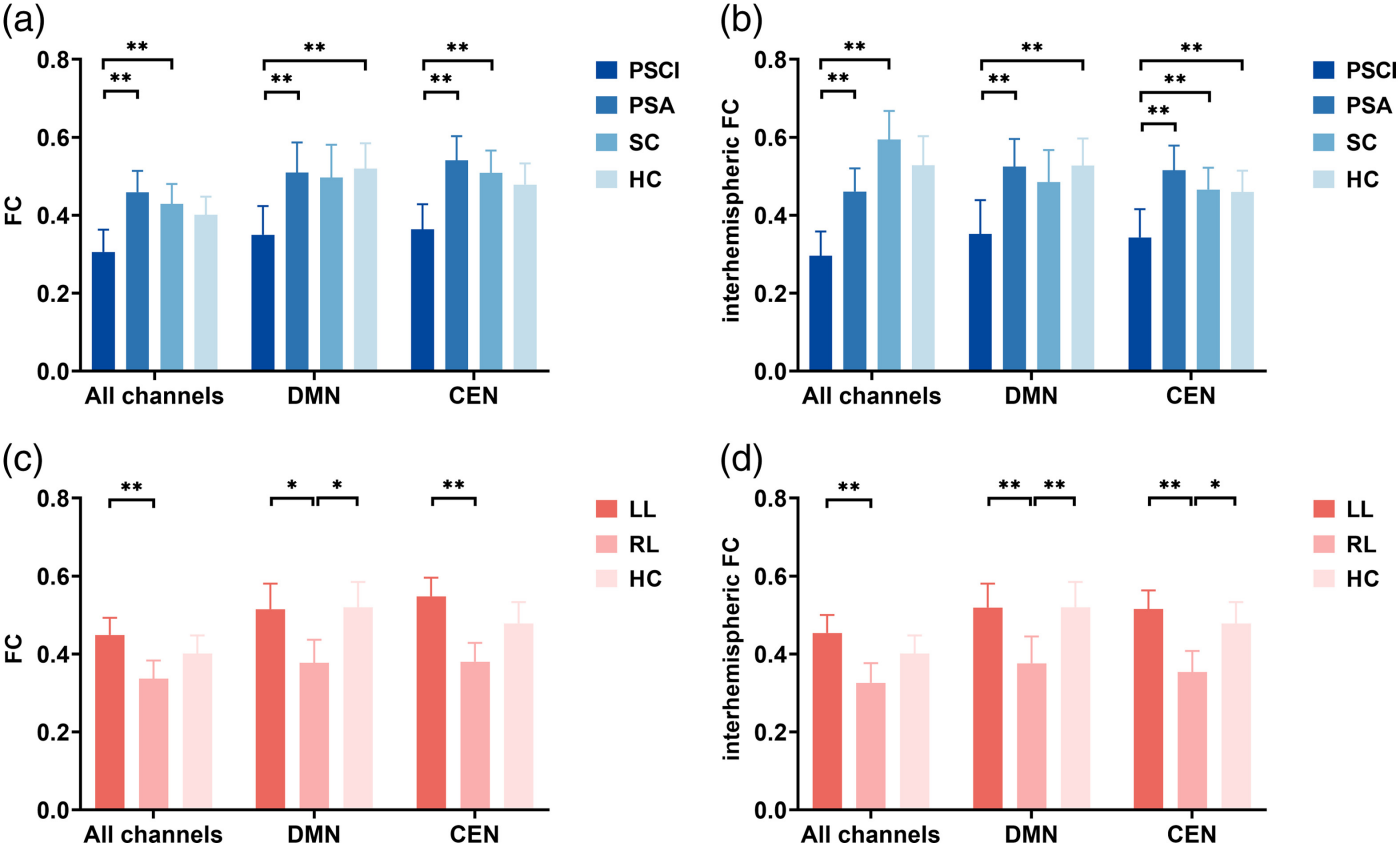
Mean and interhemispheric FC of all channels, DMN, and CEN in different groups. (a) Mean FC in different dysfunction groups, (b) interhemispheric FC in different dysfunction groups, (c) mean FC in different lesion groups, and (d) interhemispheric FC in different lesion groups. *p<0.05, **p<0.01.

We further explored the differences in the FC of brain networks in subjects with different functional disorders, as shown in Fig. 4. Post hoc comparisons revealed that the overall FC was significantly lower in the PSCI group than in the PSA ($p_{a}<0.001$) and SC ($p_{a}=0.005$) groups, but there was only marginal significance in the FC between the PSCI and HC groups ($p_{a}=0.062$). There were no significant differences between the other groups.

In addition, there were significant differences in the DMN-FC among the four groups (p=0.003). Specifically, the DMN-FC was significantly lower in PSCI patients than in PSA patients ($p_{a}=0.025$) and HCs ($p_{a}=0.008$) but marginally significantly lower than that in the SC group ($p_{a}=0.074$). Other post hoc comparisons revealed no differences.

Significant differences in the CEN-FC were also observed among the four groups (p<0.001). In addition, the CEN-FC was significantly lower in the PSCI group than in the PSA ($p_{a}<0.001$) and SC ($p_{a}=0.030$) groups. There was no significant difference between the other groups.

For interhemispheric FC, we found significant differences between patients with different types of dysfunction and healthy individuals. As shown in Fig. 4(b), the interhemispheric overall FC was significantly lower in the PSCI group than in the PSA ($p_{a}<0.001$) and SC ($p_{a}=0.005$) groups. The PSCI group had significantly lower interhemispheric DMN-FC than the PSA (PSCI versus PSA $p_{a}=0.010$) and HC (PSCI versus HC $p_{a}=0.009$) groups. Moreover, the interhemispheric CEN-FC in the PSCI group was significantly lower than that in the other three groups (PSCI versus PSA $p_{a}<0.001$; PSCI versus SC $p_{a}=0.029$; PSCI versus HC $p_{a}=0.048$).

**Fig. 5:**
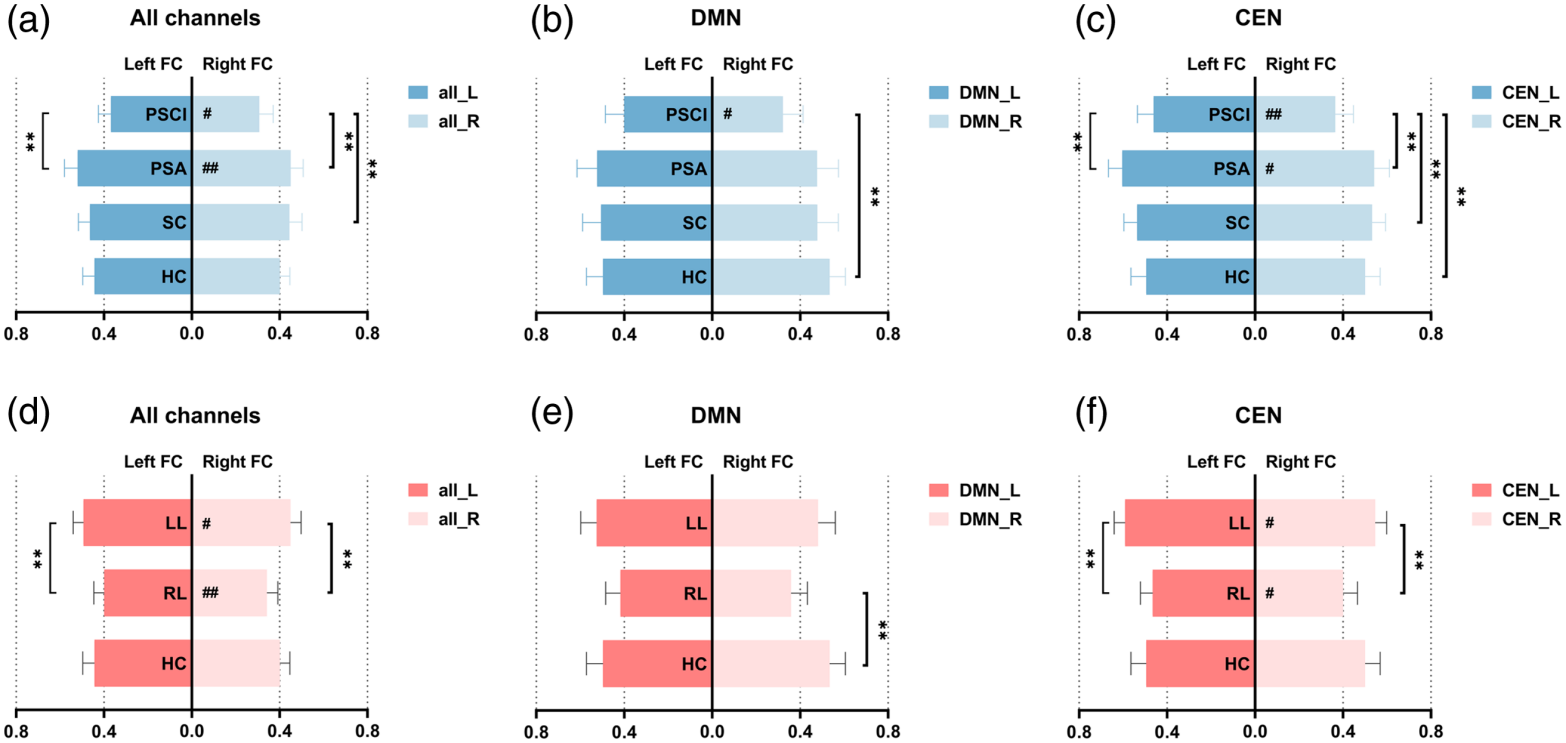
Left and right hemispherical FC of all channels, DMN, and CEN in different groups. (a)–(c) Left and right intrahemispheric FC in different dysfunction groups; (d)–(f) left and right intrahemispheric FC in different lesion groups. *p<0.05, **p<0.01 between groups; ^#^p<0.05, ^##^p<0.01 between hemispheres.

Differences were also observed in left and right intrahemispheric FCs among the different groups (Fig. 5). In the PSCI group, both the left ($p_{a}<0.001$) and right ($p_{a}=0.002$) intrahemispheric FCs were significantly lower than those in the PSA group. In addition, the right intrahemispheric FC in the PSCI group was lower than that in the SC group ($p_{a}=0.004$). Notably, there were also significant differences between the left and right intrahemispheric FCs in the PSCI (p=0.010) and PSA (p=0.001) groups, which presented greater left intrahemispheric FC [Fig. 5(a)]. By contrast, there was no significant difference between the left and right intrahemispheric FCs in the SC (p=0.310) and HC (p=0.070) groups.

The difference in bilateral CEN regions was similar to the above results; the FCs of both the left and right CENs in the PSCI group were lower than those in the PSA group, and the right FC of the CEN in the PSCI group was also lower than that in the SC and HC groups. In addition, there were significant differences in FC between the left and right CEN regions between the PSCI and PSA groups (PSCI p=0.010; PSA p=0.001) [Fig. 5(a)]. However, there was no significant difference between the other groups (SC p=0.310; HC p=0.070).

For the bilateral DMN-FCs, there was a significant difference between the left and right DMN-FCs only in the PSCI group (p=0.045) but not in the other three groups (PSA p=0.256; SC p=0.349; HC p=0.237). The right DMN-FC in the PSCI group was lower than that in the HC group ($p_{a}=0.005$), whereas there were no differences between any other groups [Fig. 5(b)].

### 3.3 Specific Changes in FC among Stroke Patients with Different Lesion Sides

We noticed that the three-stroke groups presented significant differences in the lesion side. All patients with PSA had left lesions only, whereas patients with PSCI and SC patients had a mix of left and right lesions simultaneously. Given these results, stroke patients were regrouped into left lesions (LL group, n=51) and right lesions (RL group, n=45), whereas the HC group (n=33) was used as the control to compare the influence of different lesion sides on FC.

There were significant differences between patients with left and right lesions in both overall FC, CEN-FC, and DMN-FC ($p_{\text{overall}}=0.002$; $p_{\mathrm{DMN}}=0.004$; $p_{\mathrm{CEN}}<0.001$), as shown in Fig. 4(b). In particular, post hoc comparisons revealed that the overall FC in the LL group was significantly greater than that in the RL group ($p_{a}=0.001$). DMN-FC in the RL group was significantly lower than that in the LL ($p_{a}=0.011$) and HC groups ($p_{a}=0.018$). The CEN-FC in the RL group was significantly lower than that in the LL group ($p_{a}<0.001$).

The interhemispheric overall FC between the left and right hemispheres is shown in Fig. 4(d). The interhemispheric overall FC in the RL group was lower than that in the LL group ($p_{a}<0.001$). The RL group had lower interhemispheric DMN-FC regions than the LL ($p_{a}=0.004$) and HC ($p_{a}=0.009$) groups. Moreover, the interhemispheric CEN-FC regions in the RL group were lower than that in the LL ($p_{a}<0.001$) and HC ($p_{a}=0.025$) groups.

After post hoc comparisons, we found that the FCs of the left and right hemispheres in the RL group were lower than those in the LL group (left $p_{a}=0.013$; right $p_{a}=0.004$). Moreover, the overall FC of the left hemisphere was greater than that of the right hemisphere in both the LL and RL groups ($p_{\mathrm{LL}}=0.011$; $p_{\mathrm{RL}}=0.002$). The left and right CEN-FCs in the RL group were both lower than those in the LL group (left $p_{a}=0.003$; right $p_{a}=0.001$), and the left CEN-FC was greater than CEN-FC in both the LL and RL groups ($p_{\mathrm{LL}}=0.016$; $p_{\mathrm{RL}}=0.021$). However, there was a difference in the right DMN-FC only between the RL and HC groups ($p_{a}=0.009$), and there were no differences in any of the other groups.

### 3.4 Factorial Analysis of Dysfunction or Lesion Side

As previously mentioned, we found that there were FC differences in both different dysfunctions (PSCI, PSA, and SC groups) and different lesion sides (LL and RL groups). To determine which factor plays a greater role, we performed a factorial analysis by two-way ANOVA. We first included the interaction effects of the above two factors for analysis and found that for any FC indicator, the interaction effects between the two factors were not statistically significant. Therefore, to better explore the degree of influence of these two factors, we conducted only a main effect analysis for the dysfunctions on the sides of the lesions, and the results are shown in Table 2. These results indicate that dysfunction might play a greater role in the overall FC and intrahemispheric FC, whereas the lesion side may be one of the key factors for CEN-FC.

**Table 2:** Factorial analysis of FC on the dysfunction or lesioned side.

		Overall FC	DMN- FC	CEN-FC	Left-FC	Right-FC
Dysfunction	F	3.956	2.343	3.347	3.436	3.937
	p	0.022*	0.102	0.040*	0.036*	0.023*
Lesion side	F	2.348	3.325	10.318	0.364	2.423
	p	0.129	0.071	0.003*	0.548	0.123

Overall FC represents the average FC of all channels; DMN-FC represents the average FC of the channels in the relevant regions of the DMN; CEN-FC represents the average FC of the channels in the relevant regions of the central execution network; Left-FC represents the average FC of the channels within the left hemisphere; Right-FC represents the average FC of the channels within the left hemisphere. * and **bold** represent p<0.05

### 3.5 Correlation between Behavioral Performance and FC

**Fig. 6:**
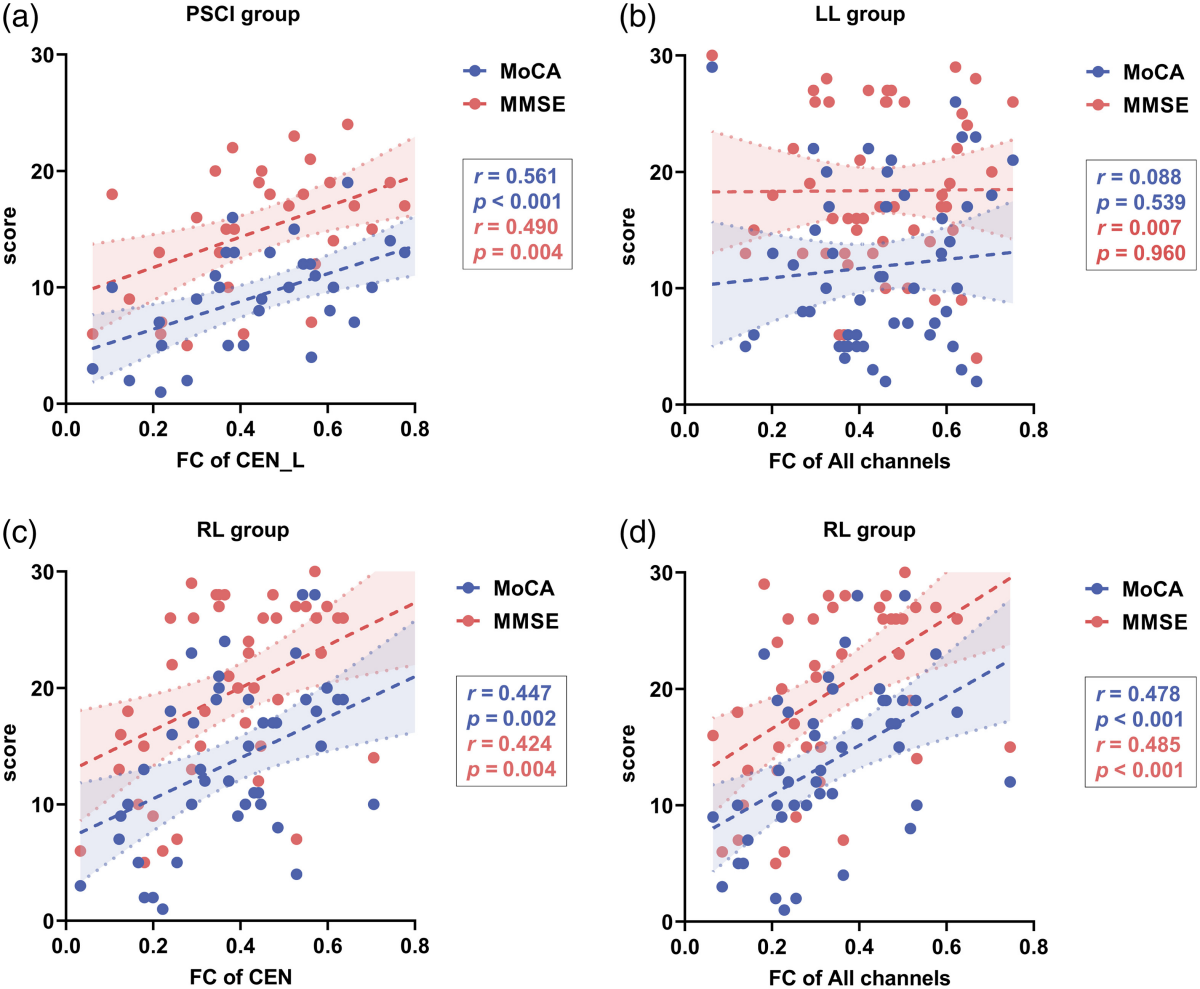
Correlations between FC and cognitive scale scores. (a) Correlations between the left CEN-FC and cognitive scores in the PSCI group; (b) correlations between overall FC and cognitive scores in the LL group; (c) correlations between the mean overall CEN-FC and cognitive scores in the RL group; (d) correlations between overall FC and cognitive scores in the RL group.

We analyzed the correlation between functional performance and FC and found that the MMSE and MoCA scores of patients in the PSCI group were significantly correlated with the left CEN-FC [Fig. 6(a)] but not with the right CEN-FC or the interhemispheric CEN-FC.

In the LL group, there was no significant correlation between scale scores and FCs [Fig. 6(b)]. In the RL group, the MMSE and MoCA scores were significantly correlated to overall FCs [Fig. 6(d)] as were the CEN-FCs [Fig. 6(c)] (overall CEN, left CEN, right CEN, and interhemispheric CEN).

## 4 Discussion and Conclusion

Our results showed that PSCI patients without aphasia had significantly lower brain FC than did normal-cognition stroke patients or healthy controls. PSA patients did not have a significant decrease in brain FC. In addition, in patients with cognitive impairment with and without aphasia, the FC of the left hemisphere is significantly greater than that of the right hemisphere, which was not significant in cognitively normal subjects.

### 4.1 Overall and Interhemispheric FC of Brain Networks Declined in PSCI Patients

Our study revealed that patients in the PSCI group without aphasia had significantly lower overall FC, DMN-FC, and CEN-FC than those in the other three groups. Moreover, cognitive performance was closely related to the CEN-FC, especially in the right hemisphere. This finding is similar to the results of some previous studies. Yue et al.^20^ also reported that in PSCI patients, interactions among the DMN, CEN, and salience network were significantly reduced. Scholars believe that the cognitive impairment of PSCI patients does not seem to have an absolute correlation with the site of injury but rather stems from the destruction of cognitive networks.^21^ Severe structural network damage leads to weakened FCs and cognitive decline. A relatively complete structural network ensures compensation for FC dysfunction, thus alleviating cognitive impairment.^22^ In addition, our previous studies confirmed the correlation between CEN-FC and cognitive recovery.^23^ However, in this study, we did not find an immediate correlation between DMN-FC and cognitive function, although many scholars believe that stroke, especially cognitive recovery, is related to DMN connectivity.^24^^,^^25^ This difference may be due to us not following up on patients to monitor subsequent changes in cognitive recovery.

On the other hand, we found that in PSCI patients, the interhemispheric overall FC was also significantly weakened. Besides, the left overall FC, CEN-FC, and DMN-FC were significantly greater than the right ones. The phenomenon of increased bilateral hemispheric independence and decreased balance after this injury has been reported many times in previous studies. The “network phenotype of stroke injury” proposed by Siegel et al.^26^ includes abnormally low interhemispheric connectivity and abnormally high intrahemispheric (ipsilateral) connectivity.^27^ Subsequent studies have shown that stroke not only damages brain networks, such as the DMN, and connectivity on the injured side, but also destroys the connectivity between brain networks on both sides.^24^ Furthermore, studies have shown that the FC consequences of stroke and neurodegenerative disease can be very different despite similar behavioral outcomes and damage to lesions. Specifically, stroke patients present asymmetric bilateral hemispheric FC changes that may lead to greater independence of hemispheric responses, whereas neurodegenerative disease may produce more symmetrical changes across hemispheres and more synchronized activity between the two hemispheres.^28^

Clinicians or therapists previously used left DLPFC and temporoparietal excitatory strategies for PSCI patients, which have achieved some improvement effects.^7^ However, our study revealed that FCs in the right hemisphere of PSCI patients were significantly lower than those in the left hemisphere and that the right CEN was closely related to cognition. These findings also suggest that the cognitive recovery of PSCI patients requires strengthening the functional activity of the whole cognitive network and promoting the balance of the bilateral hemispheres. The strategy of brain stimulation for PSCI may change from the activation of a single target to the activation of the multitarget or whole-brain network.

### 4.2 Imbalance in Bilateral FCs and Active Overall Brain Networks in PSA Patients

Notably, in our study, PSA patients did not show a significant decline in brain network FC, and the FCs within brain networks were significantly greater than those in patients with PSCI. These results may contrast with the popular assumption that dysfunction in most patients means a decline in FC, but some past research supports this view. In another fNIRS study, compared with healthy controls, patients with PSA in the subacute stage presented no difference in FC and even showed compensatory enhancement of FC in the right corresponding brain region^29^ or a massive increase in bilateral language network activation.^30^^,^^31^ In our study, the participants were all patients in the subacute phase, which may be the main reason why their FCs did not decrease.

On the other hand, in patients with PSA, we found that the overall FCs and CEN-FCs of the left hemisphere were greater than those of the right hemisphere, which is a phenomenon of bilateral brain imbalance. In fact, all lesions in these PSA patients occurred in the left hemisphere, but the left hemisphere still showed significantly greater FC than the right hemisphere. This might be a reorganizational of neuronal networks, which is interpreted as either a compensatory mechanism, reflection of inefficient neural underpinning, or a reduction in the selectivity of the supporting neural circuitry.^32^^–^^34^ The specific mechanism of the stronger FC in the PSA group is still unclear, and further studies are needed to determine. However, in this situation of overconnectivity of the left hemisphere, is activating the left hemisphere still the best treatment? Should we activate the right side of the brain to restore balance in both hemispheres?

From the previous NIBS guidelines, an excitatory stimulation in the left language area or an inhibitory stimulation in the right language mirror area have received up to a grade B recommendation, meaning that they are probably effective. However, we can also see from the guidelines that the evidence supporting this result comes from randomized controlled trials (RCTs) based on the IHI model, and only a handful of RCTs on right-hemisphere excitatory stimulation have been performed. The IHI model, an old version of the theoretical basis of stroke motor function recovery, has been challenged by compensatory recovery theory in recent decades. The bimodal balance–recovery model has gained increasing awareness, and its use in cognitive and language recovery remains to be proven.

Approximately 20 years ago, researchers postulated that right-hemisphere activation may not be useful or maybe an epiphenomenon in patients with aphasia.^35^^,^^36^ To date, there is growing evidence that the right hemisphere has a potentially beneficial role in promoting speech recovery.^37^ Imaging studies have also supported the compensatory role of the right frontal lobe in language function.^38^ Consistent with this idea, in the acute phase of aphasia, excitatory stimulation of the right hemisphere is likely to accelerate the process of speech recovery, especially in patients with left extensive brain damage.^39^ In addition, a study of combined stimulation with intonation therapy revealed that, compared with fake transcranial direct current stimulation (tDCS), anodic tDCS performed on the right inferior frontal gyrus improved speech fluency.^40^ These studies and our results suggest that right brain activation, rather than suppression, maybe a new strategy for restoring function in patients with aphasia.

### 4.3 Patients with Right-Sided Lesions Had Worse FC of the CEN, which Is Associated with Cognitive Function

In our study, we found that patients with right lesions had significantly worse FC than patients with left lesions. We hypothesize that this finding may indicate a specific compensatory increase in brain FC after left brain injury, which is consistent with the findings of PSA patients with left brain lesion. Previous studies on motor function after stroke have also revealed that right lesions lead to worse postural control, neck endurance, and recovery of activities of daily living,^41^ than left lesions do.^42^^,^^43^ Our findings are consistent with these studies. In terms of cognitive function after stroke, researchers believe that right lesions can lead to multiple deficits, especially those affecting spatial cognition.^44^ The right hemisphere plays a crucial role in maintaining directed attention and sustained attention in stroke survivors, thereby improving patients’ quality of life.^45^ The executive function of the right frontal lesion group was worse than that of the left frontal lesion group, especially in the unilateral frontal lesion group.^46^ Consistent with these findings, we suggest that compensatory elevation of brain FC in left-sided stroke patients may lead to better functional preservation and faster functional recovery. More importantly, brain networks in the right hemisphere play crucial roles in cognitive function, especially cognitive recovery, in stroke patients.

On the other hand, we also found a close correlation between cognitive performance and FC of the CEN in stroke patients with right-sided lesions, whereas no correlation between cognition and FC was found in those with left-sided lesions. This may be because left brain injury can lead to language disorders in many cases, and aphasia affects the scores of the MoCA and MMSE scales, resulting in the inability to accurately evaluate the true degree of cognitive ability of patients. In addition, we found that a specific increase in FC in patients with left lesions may lead to a mismatch between FC and behavioral performance. However, the results above suggest that functional recovery of brain networks, especially the CEN, may be the key to cognitive recovery in patients with right lesions.

## 5 Limitations

This study collected baseline data from randomized controlled studies to explore the differences in brain networks among patients with different types of cognitive impairment after stroke and to provide relevant evidence for the clinical treatment of PSCI patients. However, we failed to follow up on the subsequent rehabilitation process of the above patients and failed to analyze the correlation between functional recovery and changes in FC. This decision was made because patients receive different types of treatment, which interferes with their natural recovery. Moreover, the lack of information on small vessel disease or other underlying comorbidities at baseline may have caused some bias in the results. In addition, the gold standard of cognitive screening, the MoCA and MMSE, was selected in our study, but these scales are easily disturbed by language ability, resulting in inaccurate cognitive evaluation. In the future, an aphasia checklist and the Loewenstein Occupational Therapy Cognitive Assessment II can be used to evaluate the cognitive function of aphasia patients so that more accurate cognitive evaluations can be obtained.

## 6 Generalization

Our study was a real-world observational study that enrolled patients with various cognitive types after stroke to explore pathological changes in brain FC. Previous studies of PSCI either excluded patients with aphasia or did not distinguish them. However, language is also an important part of cognition, and patients with aphasia may have completely different pathologies and recovery mechanisms than PSCI patients without aphasia. This finding was also verified in our study.

Most previous studies of PSCI or PSA have used strategies to activate cognitive regions in the left hemisphere, such as the DLPFC or temporoparietal lobe. This is even true of some Alzheimer’s disease studies. However, our results suggest that bilateral enhanced stimulation, rather than a single stimulation of the left side, maybe a new direction for the cognitive rehabilitation of patients with PSCI. In addition, the right hemisphere may be more important to the recovery of cognition and language than previously thought, and the restoration of FC in the right hemisphere may be a key point that has been neglected. Future studies on brain stimulation can focus on the recovery of the right hemisphere, which may provide new insights into the cognitive recovery of stroke patients.

## Supplementary Material

10.1117/1.NPh.12.1.015008.s01

## Data Availability

All the data supporting the results and conclusions are included within the article or can be found at https://osf.io/fcv9n/. Raw data or other information is also available from the corresponding author/s.
