# Supplementary material for: Important role of the right hemisphere in post-stroke cognitive impairment: a functional near-infrared spectroscopy study
Source: Neurophotonics. 2025 Feb 17;12(1):015008. doi: 10.1117/1.NPh.12.1.015008 (PMC11832076; doi:10.1117/1.NPh.12.1.015008)
Supplement: Supplementary file 1 [file NPh_012_015008_SD001.pdf]

Supplemental methods

The NirSpark software (HuiChuang, China) package, which has been used in previous experiments, was used to preprocess fNIRS signals. First, an expert performed a preliminary inspection of the raw data, marking and rejecting poor-quality signals. Second, we used a spline interpolation algorithm for the resulting signals to amend motion artifacts by channels. Subsequently, further analysis of the HBO data of channels covering functionally involved areas was performed, and during preprocessing, the raw data were bandpass filtered between 0.01 and 0.2 Hz to remove physiological noise (e.g., respiration, cardiac activity, and low-frequency signal drift). The modified Beer–Lambert law was subsequently used to calculate the relative changes in HBO concentration. The FC between all 35 channels was defined as the overall FC of the brain. We combined the Brodmann and LPBA40 brain maps to divide the 35 channels into 17 regions of interest (ROIs): the left frontal pole (FP_L), right frontal pole (FP_R), left dorsolateral prefrontal cortex (DLPFC_L), right dorsolateral prefrontal cortex (DLPFC_R), Broca's area (Broca), right Broca's mirror area (Broca_M), medial prefrontal cortex (MPFC), left first motor cortex (M1_L), right first motor cortex (M1_R), left supplementary motor cortex (SMC_L), right supplementary motor cortex (SMC_R), left frontal eye fields (FEF_L), right frontal eye fields (FEF_R), left intraparietal sulus (IPS_L), right intraparietal sulus (IPS_R), left inferior parietal lobule (IPL_L) and right lateral parietal lobe (IPL_R). The average value of the FCs among the MPFC, IPL_L and IPL_R included in the default mode network (DMN) was used to represent the FC of the DMN. The mean FC of the bilateral DLPFC and bilateral IPS contained in the central executive network (CEN) was used to represent the FC of the CEN. Other brain regions or networks that are closely related to cognition or language are barely detectable on our fNIRS effective channels, so we did not analyze them. We selected 300 s after collection to analyze the FC between each brain region. Pearson correlation was used in the FC analysis, and false discovery rate (FDR) correction was performed for all FC values. In addition, we calculated the number of connected edges of each network of interest with a threshold value of 0.5.

Table S1 MNI coordinates of optrodes and channels

Optrodes/Channels	MNI coordinates		
	x	y	z
S1	58.459	-12.381	51.335
S2	58.808	31.715	2.1472
S3	30.957	66.062	6.7866
S4	-22.302	70.493	10.631
S5	-59.224	30.897	9.7542
S6	-51.545	-34.218	61.001
S7	39.044	-14.556	70.224
S8	44.16	44.488	30.407
S9	7.0939	61.85	35.915
S10	-38.376	45.124	35.146
S11	-43.601	10.797	59.43
S12	15.785	-14.29	77.415
S13	-31.399	-32.447	75.252
S14	-23.676	10.494	70.8
D1	45.018	10.621	57.928
D2	48.45	52.269	4.3486
D3	7.1271	72.606	9.1434
D4	-45.552	54.986	11.089
D5	-58.352	-12.496	52.027
D6	51.459	-34.471	61.033
D7	56.726	26.182	26.023
D8	27.143	57.465	32.916
D9	-17.153	57.861	36.809
D10	-52.61	26.207	38.338
D11	-37.627	-12.711	70.059
D12	27.055	-35.401	76.297
D13	24	7.9892	71.288
D14	-16.716	-12.258	77.544
CH1 (S1-D1)	52.356	-1.9313	55.131
CH2 (S1-D6)	56.299	-23.562	56.601
CH3 (S2-D2)	55.399	41.65	1.9866
CH4 (S2-D7)	59.693	29.199	14.255
CH5 (S3-D2)	41.854	60.848	5.2035
CH6 (S3-D3)	19.831	71.754	8.2877
CH7 (S3-D8)	29.242	63.37	19.568
CH8 (S4-D3)	-10.758	73.07	10.168
CH9 (S4-D4)	-35.079	62.495	11.108
CH10 (S4-D9)	-21.034	65.834	24.694
CH11 (S5-D4)	-52.275	42.85	10.496
CH12 (S5-D10)	-56.346	28.713	24.181

CH13 (S6-D5)	-55.973	-23.32	57.463
CH14 (S6-D11)	-45.237	-23.017	66.994
CH15 (S7-D1)	42.319	-2.2922	62.568
CH16 (S7-D6)	45.142	-23.342	67.067
CH17 (S7-D12)	33.732	-24.261	73.163
CH18 (S7-D13)	30.879	-4.5054	68.988
CH19 (S8-D2)	46.574	49.425	18.266
CH20 (S8-D7)	51.663	35.728	29.39
CH21 (S8-D8)	36.333	51.127	30.619
CH22 (S9-D3)	6.9278	68.503	23.942
CH23 (S9-D8)	16.238	59.508	34.233
CH24 (S9-D9)	-8.4826	60.969	37.456
CH25 (S10-D4)	-42.728	50.755	24.327
CH26 (S10-D9)	-29.041	53.06	37.191
CH27 (S10-D10)	-45.617	35.222	37.851
CH28 (S11-D5)	-50.878	-2.2923	56.073
CH29 (S11-D11)	-40.425	-0.87957	63.65
CH30 (S12-D12)	20.744	-25.16	77.428
CH31 (S12-D13)	20.445	-2.6141	76.598
CH32 (S13-D11)	-34.819	-22.105	73.515
CH33 (S13-D14)	-23.263	-20.709	77.36
CH34 (S14-D11)	-30.534	-1.1513	68.7
CH35 (S14-D14)	-19.211	-0.49547	76.226
